# Doxorubicin inhibits osteosarcoma progression by regulating circ_0000006/miR-646/ BDNF axis

**DOI:** 10.1186/s13018-021-02782-y

**Published:** 2021-10-30

**Authors:** Abulimiti Amuti, Dehu Liu, Ayiguli Maimaiti, Yao Yu, Yalikun Yasen, Haoguang Ma, Rui Li, Shurong Deng, Fei Pang, Youliang Tian

**Affiliations:** 1 Department of Orthopaedics, The Second Affiliated Hospital of Xinjiang Medical University, Urumqi, Xinjiang China; 2 Department of Osteology, Tai’an Traditional Chinese Medicine Hospital, Taian, Shandong China; 3 Six Subjects of Hand Surgery, Affiliated Central Hospital of Shenyang Medical College, Shenyang, Liaoning China; 4 Department of Surgery, Hot Spring Sanatorium of Linyi, Linyi Hedong Central Hospital, Linyi, Shandong China; 5 Department of Joint Surgery, The Fourth Hospital of Baotou, Baotou City, Mongolia China; 6 Department of Pharmacy, The First Affiliated Hospital of Jinan University, Guangzhou, Guangdong China; 7 Department of Orthopaedics, Shaoxing People’s Hospital, No. 568 North Zhongxing Road, Yuecheng District, Shaoxing City, 312000 Zhejiang Province China; 8 Department of Rehabilitation Medicine and Physiotherapy, PLA Strategic Support Force Characteristic Medical Center, No. 9 Anxiang North Lane, Chaoyang District, , Beijing, 100101 China

**Keywords:** DOX, circ_0000006, miR-646, BDNF, Osteosarcoma

## Abstract

**Background:**

Osteosarcoma (OS) is the most common aggressive bone tumor in children and teenagers. Doxorubicin (DOX) is a chemotherapeutic drug for OS. This study aims to reveal the effects and underneath mechanism of DOX treatment in OS progression.

**Methods:**

The expression of circular_0000006 (circ_0000006), microRNA-646 (miR-646) and brain-derived neurotrophic factor (BDNF) was detected by quantitative real-time polymerase chain reaction (qRT-PCR). BDNF protein expression was determined by western blot. Cell proliferation was illustrated by cell counting kit-8 (CCK-8) and cell colony formation assays. Cell migration and invasion were revealed by transwell migration and wound-healing assays and transwell invasion assay, respectively. Cell apoptosis was demonstrated by flow cytometry analysis. The binding relationship of miR-646 and circ_0000006 or BDNF was predicted by circRNA interactome and targetscan online database, respectively, and verified by dual-luciferase reporter assay. The effects of circ_0000006 knockdown on tumor growth in vivo were manifested by in vivo tumor formation assay.

**Results:**

Circ_0000006 expression and the mRNA and protein levels of BDNF were dramatically upregulated, and miR-646 expression was effectively downregulated in OS tissues or cells compared with control groups. Circ_0000006 expression and BDNF protein expression were lower, and miR-646 expression was higher in DOX treatment groups than in control groups in OS cells. Circ_0000006 knockdown repressed cell proliferation, migration and invasion, whereas promoted cell apoptosis under DOX treatment in OS cells; however, these effects were attenuated by miR-646 inhibitor. Additionally, circ_0000006 sponged miR-646 to bind to BDNF. Circ_0000006 silencing suppressed tumor growth in vivo.

**Conclusion:**

Circ_0000006 knockdown promoted DOX-mediated effects on OS development by miR-646/BDNF pathway, which provided a theoretical basis in treating OS with DOX.

## Introduction

Osteosarcoma (OS) is a prime bone tumor caused by mesenchymal cells in children and teenagers [1, 2]. The fatality ratio of OS is violently high and more than 20% cases survive less than 5 years [3]. OS treatment is frequently defeated by the strong abilities of OS in metastasis and proliferation [4, 5]. Doxorubicin (DOX) is chosen as the first-line drug in treating OS [6]. But DOX is inoperative and results in drug resistance at a low dose and is highly toxic at a high dose [7]. Therefore, exploring the mechanism of DOX treatment in OS progression is urgent for its clinical application.

Circular RNA (circRNA) is a noncoding RNA with a closed continuous loop, thereby more stable than linear RNA [8, 9]. CircRNA is generated by back-splicing from pre-mRNA transcripts and widely expressed in the transcripts of eucaryon [10, 11]. Studies have indicated circRNAs are linked to cancer process [12, 13]. For example, Jian et al. illustrated that circ_001680 could modulate cell proliferative and migratory abilities via sponging microRNA-340 (miR-340) in colorectal carcinoma [14]. Yang et al. revealed that circ_0046264 inhibited lung cancer process by binding to miR-1245 [15]. Circ_0007534 was also reported to participate in the apoptosis of OS cells [16]. In addition, circRNAs are involved in DOX chemosensitivity in cancer cells. For instance, Hu et al. unveiled that circ_101057 accelerated DOX sensitivity in OS [17]. From the study of Ji et al., we found that the difference in circ_0000006 expression was the most significant among ten dysregulated circRNAs in 30 pairs of OS tissues, as compared with paired adjacent tissues; also, the study revealed that circ_0000006 contributed to OS malignant progression [18]. However, there are no data on the function of circ_0000006 regulating DOX-mediated OS progression.

MiRNA is a small noncoding RNA, harboring about 20 nucleotides (nts) [19]. MiRNA plays a vital part in cancer progression and can repress or promote cancer process [20, 21]. Dai et al. demonstrated that miR-646 suppressed the proliferative and migratory abilities of colorectal cancer cells [22]. Zhang et al. disclosed that miR-646 hindered cell proliferation and tumor metastasis in gastric cancer [23]. Nevertheless, the regulatory mechanism of miR-646 in OS development is still unclear.

Brain-derived neurotrophic factor (BDNF), a neural factor, has been revealed to act as an oncogene [24]. BDNF was displayed to accelerate cell migratory and invasion in ovarian [25] and cervical cancer [26]. Zhang et al. found the low expression of BDNF inhibited cell proliferation and invasion in lung cancer [27]. These data suggest that BDNF may be related to OS progression.

Herein, the expression of circ_0000006, miR-646 and BDNF was determined in OS tissues, cells and DOX-mediated OS cells. The influences of DOX on the cell proliferation, migration, invasion and apoptosis were revealed. Furthermore, whether the regulatory mechanism of DOX in OS development was attributed to circ_0000006/miR-646/BDNF pathway was unveiled.

## Materials and methods

### Specimen collection

Twenty pairs of OS and normal bone tissues were obtained from OS patients from the Second Affiliated Hospital of Xinjiang Medical University. Entire tissues were kept at − 80 °C in a freezer for subsequent experiment. The Ethics Committee of the Second Affiliated Hospital of Xinjiang Medical University consented this experiment. OS patients related to this study wrote the informed consents. All subjects were not treated with neoadjuvant chemotherapy.

### Cell acquisition and culture

Otwobiotech (Shenzhen, China) provided human OS cell lines (U2OS and SJSA1) and human fetal osteoblast cell line hfOB 1.19. Cells were grown in Dulbecco’s modified Eagle’s medium (DMEM; Thermo Fisher, Waltham, MA, USA) supplementing with 10% fetal bovine serum (FBS; Thermo Fisher) with 1% streptomycin/penicillin (Thermo Fisher). U2OS and SJSA1 cells were cultured at 37 °C, and hfOB 1.19 cells were grown at 34 °C in an incubator with 5% CO_2_.

### Cell transfection

Small inferring RNA targeting circ_0000006 (si-circ_0000006), small hairpin RNA against circ_0000006 (sh-circ_0000006), miR-646 mimic, miR-646 inhibitor, the overexpression plasmid of BDNF (pc-BDNF) and controls (si-NC, sh-NC, miRNA NC, inhibitor NC and pc-NC) were amplified by GenePharma (Shanghai, China). Lipofectamine 2000 was employed to transfect plasmids or fragments into U2OS and SJSA1. The synthesized sequences in this part were si-circ_0000006 5′-CTTTACGGACGTCCCAGTGAT-3′, miR-646 mimic 5′-AAGCAGCUGCCUCUGAGGC-3′, miR-646 inhibitor 5′-GCCUCAGAGGCAGCUGCUU-3′, si-NC 5′-CCTCTACCTGTCGCTGAGCTGTAAT-3′, miRNA NC 5′-UUUGUACUACACAAAAGUACUG-3′ and inhibitor NC 5′-CAGUACUUUUGUGUAGUACAAA-3′.

### Quantitative real-time polymerase chain reaction (qRT-PCR)

OS tissues and cells were lysed with TRIzol reagent (TaKaRa, Dalian, China). RNA concentration and purity were detected via NanoDrop-1000 apparatus (Thermo Fisher). cDNA was synthesized with PrimeScript RT Master Mix (TaKaRa) and miRNA reverse transcription kit (Thermo Fisher). For unveiling the expression of circ_0000006, miR-646 and BDNF, SYBR Green SuperMix (Corning, New York, Madison, USA) was performed with Mx3000P system (Stratagene, Santa Clara, CA, USA). Data were analyzed with the 2^−∆∆Ct^ method. U6 and glyceraldehyde 3-phosphate dehydrogenase (GAPDH) were selected as controls. The sense and anti-sense primers were circ_0000006 5′-GCACTGTCCACCAACATCA-3′ and 5′-AAGCTCTTCCCGCTCCTC-3′; miR-646 5′-AGCAGCTGCCTCTGAG-3′ and 5′-GAACATGTCTGCGTATCTC-3′; BDNF 5′-GTTTGTGTGGACCCCGAGTT-3′ and 5′-CCACCTTGTCCTCGGATGTT-3′; U6 5′-CTCGCTTCGGCAGCACA-3′ and 5′-AACGCTTCACGAATTTGCGT-3′; GAPDH 5′-CTCTGCTCCTCCTGTTCGAC-3′ and 5′-GCGCCCAATACGACCAAATC-3′.

### RNase R treatment assay

U2OS and SJSA1 cells were lysed with TRIzol reagent (TaKaRa), and RNA was extracted with EasyPure® RNA Kit (TransGen Biotech, Beijing, China). Two μg RNA was incubated with or without 5 U RNase R (Epicentre, Madison, WI, USA) at 37 °C for 30 min, respectively. And RNA was purified using RNeasy MinElute Cleaning Kit (Qiagen, Valencia, CA, USA). QRT-PCR was performed to determine the expression of circ_0000006 and GAPDH mRNA.

### Cell viability assay

Cell Counting Kit-8 (CCK-8; Beyotime, Jiangsu, China) was purchased to detect the viability of U2OS and SJSA1 cells. In short, cells were grown in 96-well plates (5000 cells per well) and cultured for 24 h. Following that, cells were disposed with obvious treatments after DOX (Beyotime) exposure. 20 μL CCK-8 solution (Beyotime) was incubated with cells for 2 h. Cell viability was revealed by measuring absorbance at 450 nm using microplate reader (Thermo Labsystems, Waltham, MA, USA).

### Transwell migration and invasion assays

The migration and invasion of U2OS and SJSA1 cells were determined with transwell chamber without or with Matrigel (Corning), respectively. 1 × 10^5^ cells were cultured in the upper chamber in FBS-free DMEM (Thermo Fisher) for each experiment. DMEM containing 20% FBS (Thermo Fisher) was added into the lower chamber. Twenty-four hours later, medium was removed and cells were washed with phosphate buffer solution (PBS; Thermo Fisher). After that, cells were incubated with methanol (Beyotime) and crystal violet (Beyotime). Results were analyzed under microscope (Olympus, Tokyo, Japan) at a 100 magnification.

### Cell colony formation assay

U2OS and SJSA1 cells were cultivated in 6-well plates (500 cells each well) for 2 weeks after various treatments. DMEM medium (Thermo Fisher) was changed every 3 days. Medium was removed, and proliferative colonies were incubated with paraformaldehyde (Beyotime) and dyed with crystal violet (Beyotime). The numbers of colonies were calculated under microscope (Olympus). A colony was regarded when its cell numbers reached 50.

### Wound-healing assay

U2OS and SJSA1 cells were treated and grown in 6-well plates. Wounds were made with pipette tips when the confluence of cells reached 100%. FBS-free DMEM (Thermo Fisher) was added into the plates and cells were cultured for 24 h. The area occupied by migratory cells was calculated via microscope (Olympus) with 100 × magnification.

### Flow cytometry analysis

Annexin V-fluorescein isothiocyanate (Annexin V-FITC)/propidium iodide (PI) detection kit (Yeasen Biotech, Shanghai, China) was performed to determine cell apoptosis. In short, cells were digested and harvested at 48 h after treatment. Cells were washed with pre-cold PBS (Thermo Fisher) and centrifuged at 200 rpm for 5 min. Cells were resuspended in 100 μL binding buffer (Yeasen Biotech). Following that, cells were incubated with 5 μL Annexin V-FITC (Yeasen Biotech) and PI (Yeasen Biotech) for 12 min in dark. Results were assessed via flow cytometry (BD Biosciences, San Diego, CA, USA).

### Dual-luciferase reporter assay

The binding sites of miR-646 and circ_0000006 or BDNF were predicted by circRNA interactome or targetscan online database. The wild type (WT) of circ_0000006 and BDNF 3′-untranslated regions (3′UTR) containing the binding sequences of miR-646 were cloned into pmirGLO vector (Promega, Madison, WI, USA), and named as WT-circ_0000006 and WT-BDNF 3′UTR, respectively. The sites bound by miR-646 in circ_0000006 and BDNF 3′UTR were mutated and mutant (MUT) circ_0000006 and BDNF 3′UTR were inserted into pmirGLO vector (Promega), and called as MUT-circ_0000006 and MUT-BDNF 3′UTR. Plasmids were transfected into U2OS and SJSA1 cells with miR-646 mimic or miRNA NC using DharmaFECT 4 (Thermo Fisher). Luciferase activities were disclosed via dual-luciferase reporter assay kit (Promega) with *Renilla* Luciferase as a control.

### Western blot analysis

OS tissues and cells were lysed with RIPA buffer (Beyotime). Lysate was boiled in boiling water for 8 min and suspended in loading buffer (Solarbio, Beijing, China). Protein sample was loaded by 12% sodium dodecyl sulfonate-polyacrylamide gel electrophoresis (SDS-PAGE; Beyotime). Protein bands were transduced onto nitrocellulose membranes (GE Healthcare, Westborough, MA, USA) and incubated in 5% nonfat milk (Solarbio) at 4 °C for 5 h. After that, membranes were incubated with anti-BDNF (1:1000; CST, Boston, MA, USA) and anti-GAPDH (1:1000; CST) at 4 °C overnight. The membranes were incubated with secondary antibody marked horseradish peroxidase (1:2000; CST) at 37 °C for 2 h. Protein bands were visualized under enhanced chemiluminescence (KeyGen, Nanjing, China). GAPDH was employed as a reference.

### In vivo tumor formation assay

Charles River (Beijing, China) furnished BALB/c nude mice (5-week old). Nude mice were grown in pathogen-free conditions. Nude mice were divided into two groups (*N* = 6 per group). U2OS cells (1 × 10^6^) stably transfected with sh-circ_0000006 or sh-NC were hypodermically injected into nude mice. Seven days later, tumor volume was measured every 1 week. All mice were killed after 28 days and the size and weight of tumor were detected. A part of each tumor was excised and stored at − 80 °C for analysis of circ_0000006, miR-646 and BDNF expression. The Animal Care Committee of the Second Affiliated Hospital of Xinjiang Medical University agreed with this part.

### Statistical analysis

Every experiment was repeated at least 3 times. SPSS 21.0 software (IBM, Somers, NY, USA) was employed to analyze data. Value was shown as means ± standard deviations. Pairwise differences between groups were assessed by two-tailed Student’s *t* tests. *P* value < 0.05 was considered statistically significant (*P < 0.05 and **P < 0.01).

## Results

### Circ_0000006 expression was dramatically downregulated by DOX exposure in OS cells

**Fig. 1:**
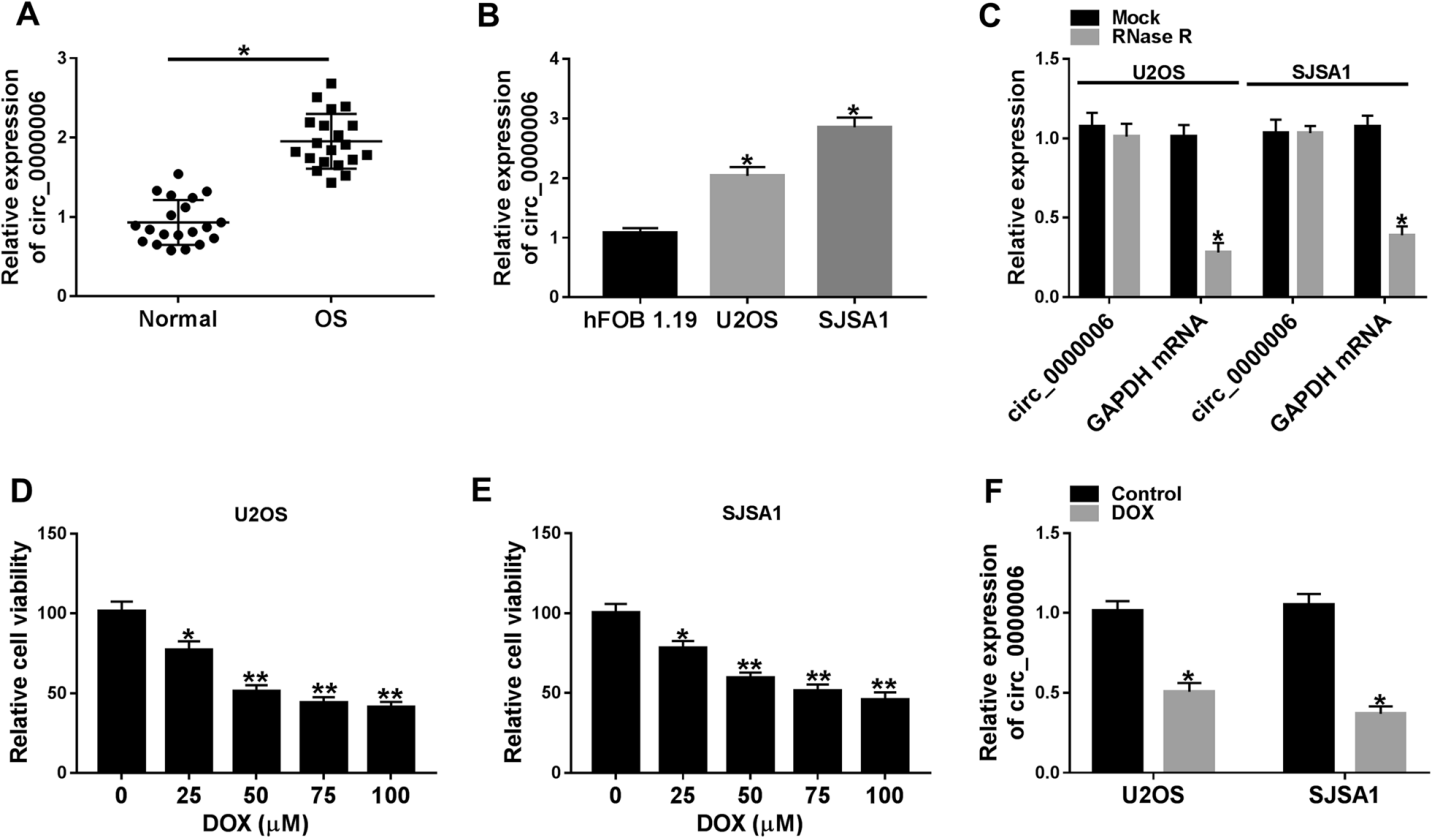
Circ_0000006 was lowly expressed after DOX treatment in OS cells. **A**, **B** QRT-PCR was performed to detect circ_0000006 expression in 20 pairs of OS and normal bone tissues and hFOB 1.19, U2OS and SJSA1 cells. **C** RNase R treatment assay was employed to illustrate that circ_0000006 was a circular RNA. **D**, **E** Cell viability assay was used to determine the optimum concentration of DOX in U2OS and SJSA1 cells. **F** The effect of DOX exposure on circ_0000006 expression was revealed by qRT-PCR in U2OS and SJSA1 cells. *P < 0.05 and **P < 0.01

In order to illustrate the characteristic of circ_0000006 in DOX-mediated OS progression, its expression was firstly determined in OS tissues and cells. Results showed that circ_0000006 expression was remarkably upregulated in OS tissues (*N* = 20) and U2OS and SJSA1 cells relative to normal bone tissues (*N* = 20) and hFOB 1.19 cells, respectively (Fig. 1A, B). Subsequently, RNase R treatment assay manifested that circ_0000006 expression had no dramatic change after RNase R treatment in U2OS and SJSA1 cells, whereas GAPDH mRNA level was obviously downregulated after RNase R exposure (Fig. 1C), which suggested that circ_0000006 was a circular RNA. Furthermore, cell viability assay demonstrated that DOX (25, 50, 75 and 100 μM) repressed cell viability in a dose-dependent manner in U2OS and SJSA1 cells; however, cell viability could not be reduced further when DOX concentration more than 50 µM (Fig. 1D, E), and this concentration was chosen for subsequent experiments. QRT-PCR results testified DOX exposure dramatically suppressed circ_0000006 expression in U2OS and SJSA1 cells (Fig. 1F). These data suggested that DOX might acted as a tumor suppressor by regulating circ_0000006 expression in OS development.

### Circ_0000006 knockdown enhanced DOX-mediated impacts on OS progression

**Fig. 2:**
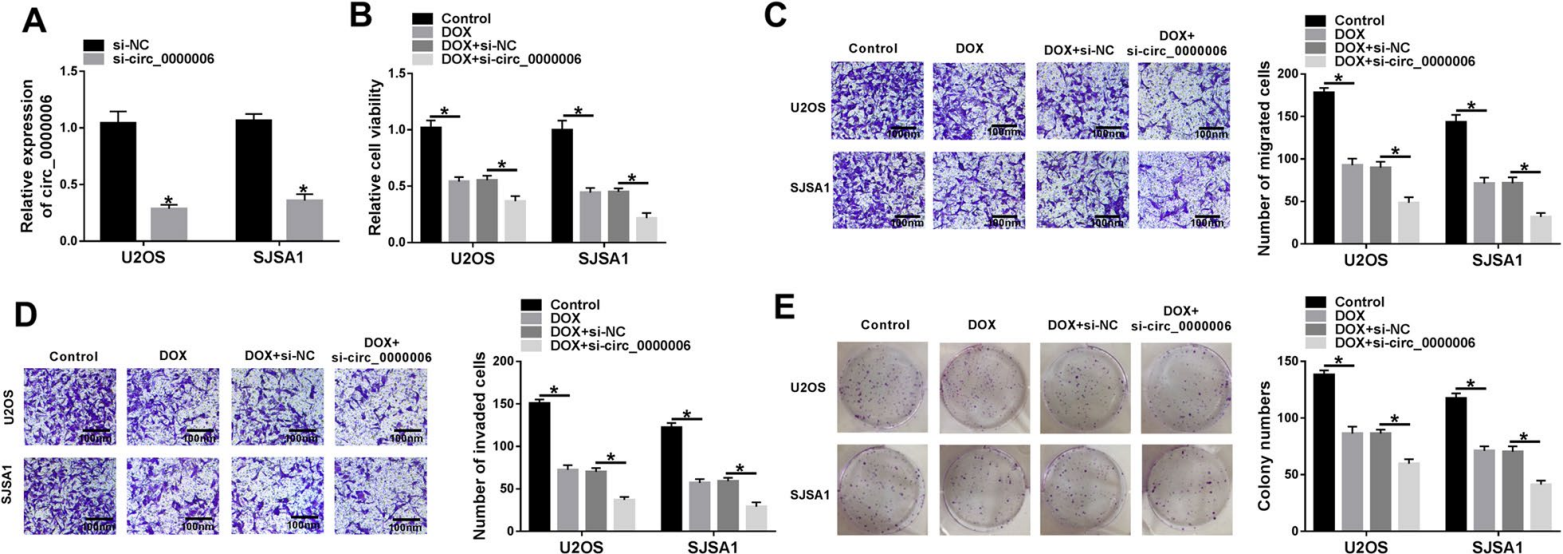
DOX exposure inhibited cell proliferation, migration and invasion, and circ_0000006 knockdown contributed to these effects in OS cells. **A** Circ_0000006 expression was determined after circ_0000006 knockdown by qRT-PCR in U2OS and SJSA1 cells. **B**, **E** Cell viability and cell colony formation assays were performed to reveal the effects between DOX treatment and circ_0000006 depletion on cell proliferation in U2OS and SJSA1 cells. **C**, **D** Transwell migration and invasion assays were performed to testify the impacts between DOX exposure and circ_0000006 depletion on the migration and invasion of U2OS and SJSA1 cells. *P < 0.05

**Fig. 3:**
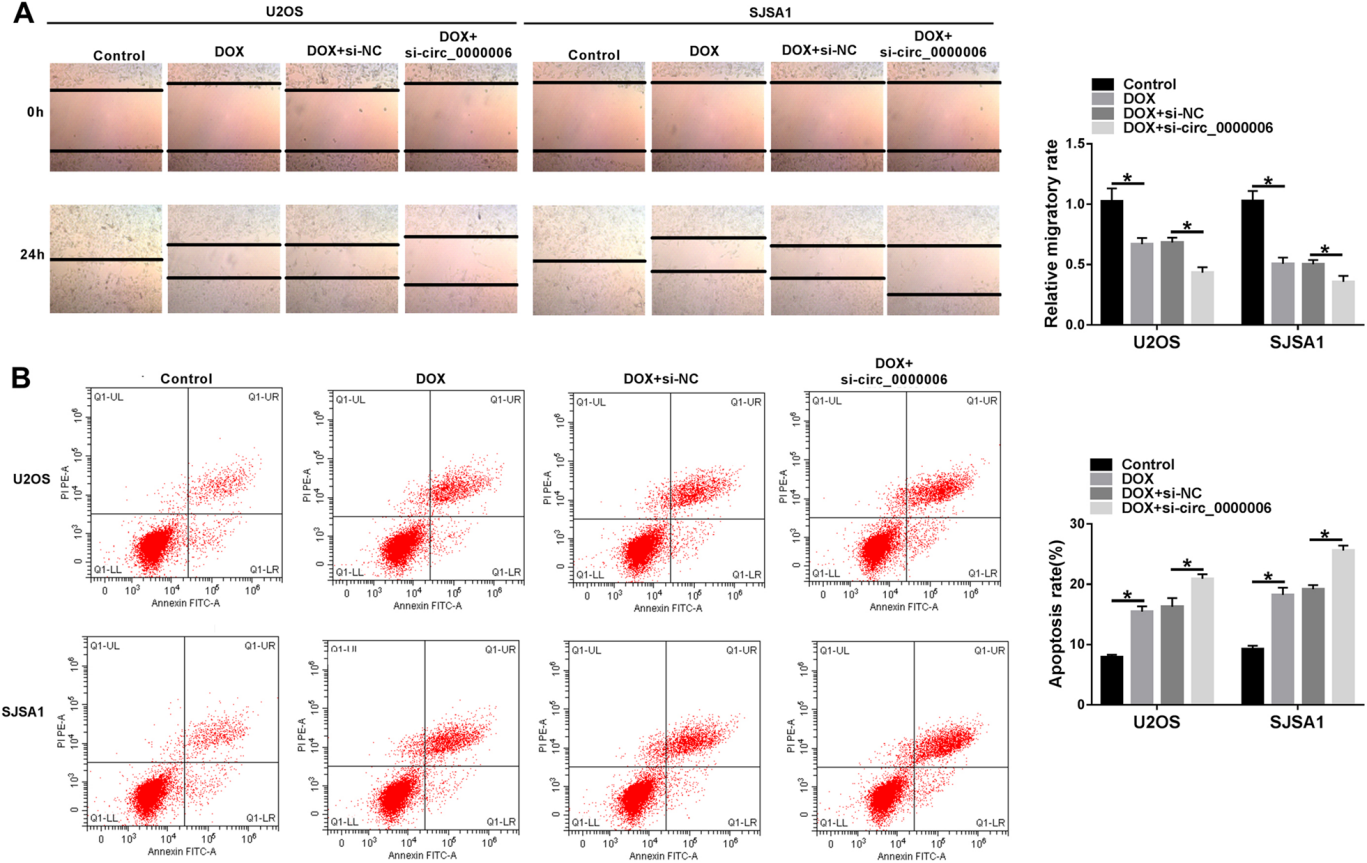
DOX treatment inhibited cell migration and induced cell apoptosis, and circ_0000006 silencing expedited these influences in OS cells. **A** The impacts between DOX treatment and circ_0000006 repression on cell migration were unveiled by wound-healing assay in U2OS and SJSA1 cells. **B** Flow cytometry analysis was carried out to investigate the influences between DOX treatment and circ_0000006 depletion on the apoptosis of U2OS and SJSA1 cells. *P < 0.05

The effects between DOX and circ_0000006 silencing on OS development were studied. The efficiency of circ_0000006 knockdown was firstly determined in U2OS and SJSA1 cells. Result displayed that circ_0000006 expression was significantly repressed after si-circ_0000006 transfection (Fig. 2A). Subsequently, cell viability assay elucidated that DOX exposure repressed cell viability and circ_0000006 knockdown enhanced this effect in U2OS and SJSA1 cells (Fig. 2B). It was found that DOX treatment inhibited cell migration and invasion, respectively, and circ_0000006 depletion promoted these inhibitory impacts in U2OS and SJSA1 cells (Figs. 2C, D, 3A). Cell colony formation assay also unveiled that DOX exposure restrained cell colony-forming ability, and this influence was facilitated after si-circ_0000006 transfection in U2OS and SJSA1 cells (Fig. 2E). Additionally, flow cytometry analysis manifested that the apoptosis of U2OS and SJSA1 cells was induced by DOX treatment, and circ_0000006 repression expedited this impact (Fig. 3B). All above results corroborated that circ_0000006 silencing repressed the resistance of U2OS and SJSA1 cells to DOX.

### Circ_0000006 acted as a sponge of miR-646 in OS cells

**Fig. 4:**
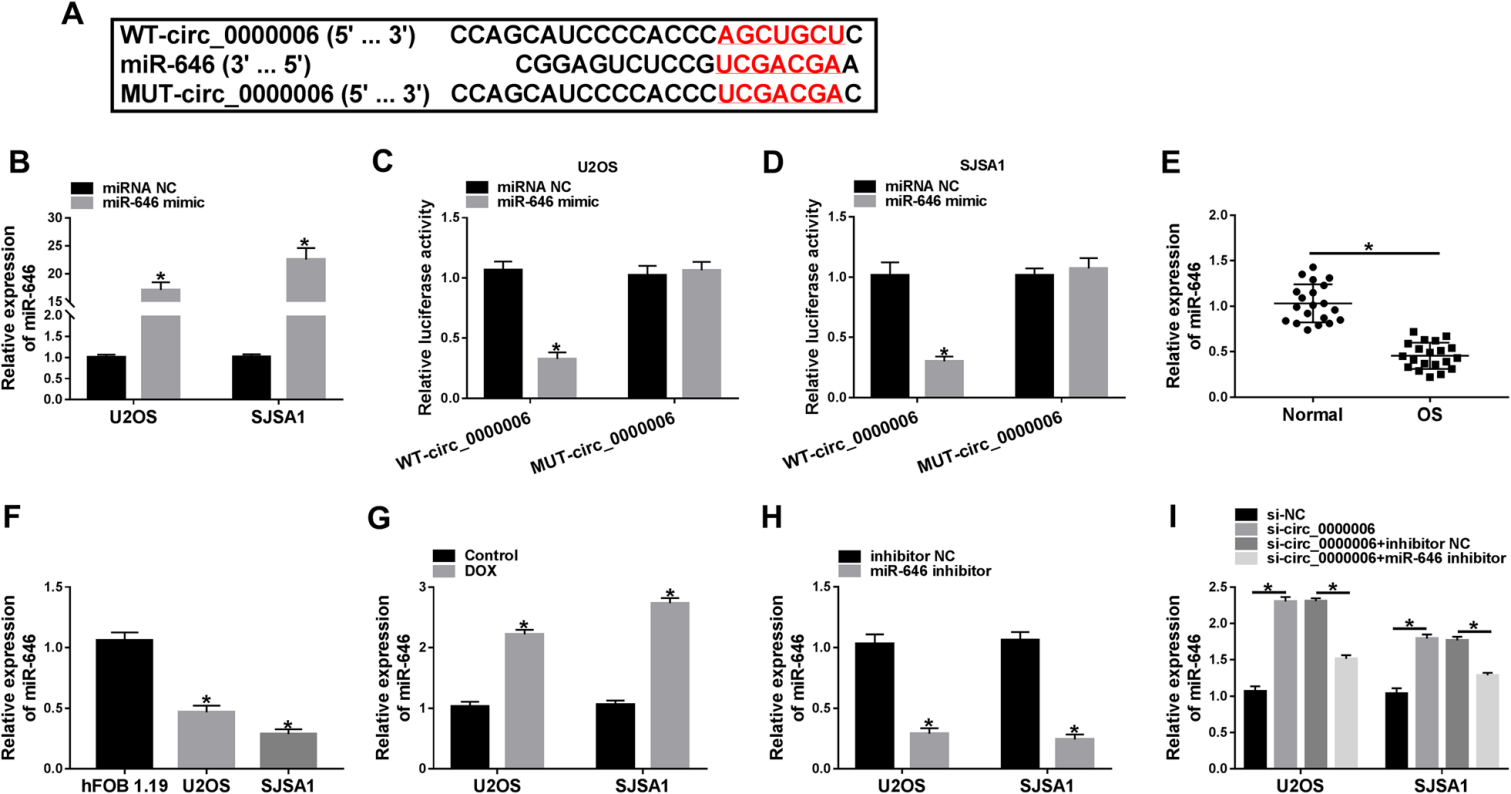
Circ_0000006 bound to miR-646 in U2OS and SJSA1 cells. **A** The binding sites between circ_0000006 and miR-646 were predicted by circRNA interactome online database. **B**, **H** The efficiency of miR-646 mimic and inhibitor in increasing or decreasing miR-646 expression was detected by qRT-PCR in U2OS and SJSA1 cells. **C**, **D** Dual-luciferase reporter assay was employed to determine luciferase activities in U2OS and SJSA1 cells. **E**, **F** QRT-PCR was performed to detect miR-646 expression in normal bone tissues, OS tissues and hFOB 1.19, U2OS and SJSA1 cells. **G** The effect of DOX treatment on miR-646 expression was illustrated by qRT-PCR in U2OS and SJSA1 cells. **I** QRT-PCR was carried out to demonstrate the effects between circ_0000006 knockdown and miR-646 depletion on miR-646 expression in U2OS and SJSA1 cells. *P < 0.05

In order to reveal the regulatory mechanism of circ_0000006 in DOX-mediated OS process, circRNA interactome online database was employed. Result disclosed that circ_0000006 contained the binding sites of miR-646 (Fig. 4A). To further testify the binding relationship between circ_0000006 and miR-646, the efficiency of miR-646 overexpression was primarily determined. Results showed miR-646 expression was strikingly upregulated by miR-646 mimic in U2OS and SJSA1 cells (Fig. 4B). Dual-luciferase reporter assay illustrated that the luciferase activity of WT-circ_0000006 + miR-646 mimic group was notably repressed in U2OS and SJSA1 cells, whereas there was no prominent change in MUT-circ_0000006 + miR-646 mimic group (Fig. 4C, D). Subsequently, qRT-PCR results demonstrated that miR-646 expression was effectively downregulated in OS tissues and U2OS and SJSA1 cells as compared to normal bone tissues and hFOB 1.19 cells, respectively (Fig. 4E, F). Additionally, miR-646 expression was upregulated by DOX treatment in U2OS and SJSA1 cells (Fig. 4G). In order to explain the effects between circ_0000006 knockdown and miR-646 inhibitor on miR-646 expression, the efficiency of miR-646 silencing was incipiently determined. QRT-PCR analysis displayed that miR-646 expression was apparently inhibited by miR-646 inhibitor in U2OS and SJSA1 cells (Fig. 4H). Subsequent data unveiled that miR-646 expression was upregulated by circ_0000006 silencing, whereas miR-646 inhibitor attenuated this effect in U2OS and SJSA1 cells (Fig. 4I). All data demonstrated that circ_0000006 was associated with miR-646 in U2OS and SJSA1 cells.

### Circ_0000006 knockdown contributed to DOX-mediated OS progression by sponging miR-646

**Fig. 5:**
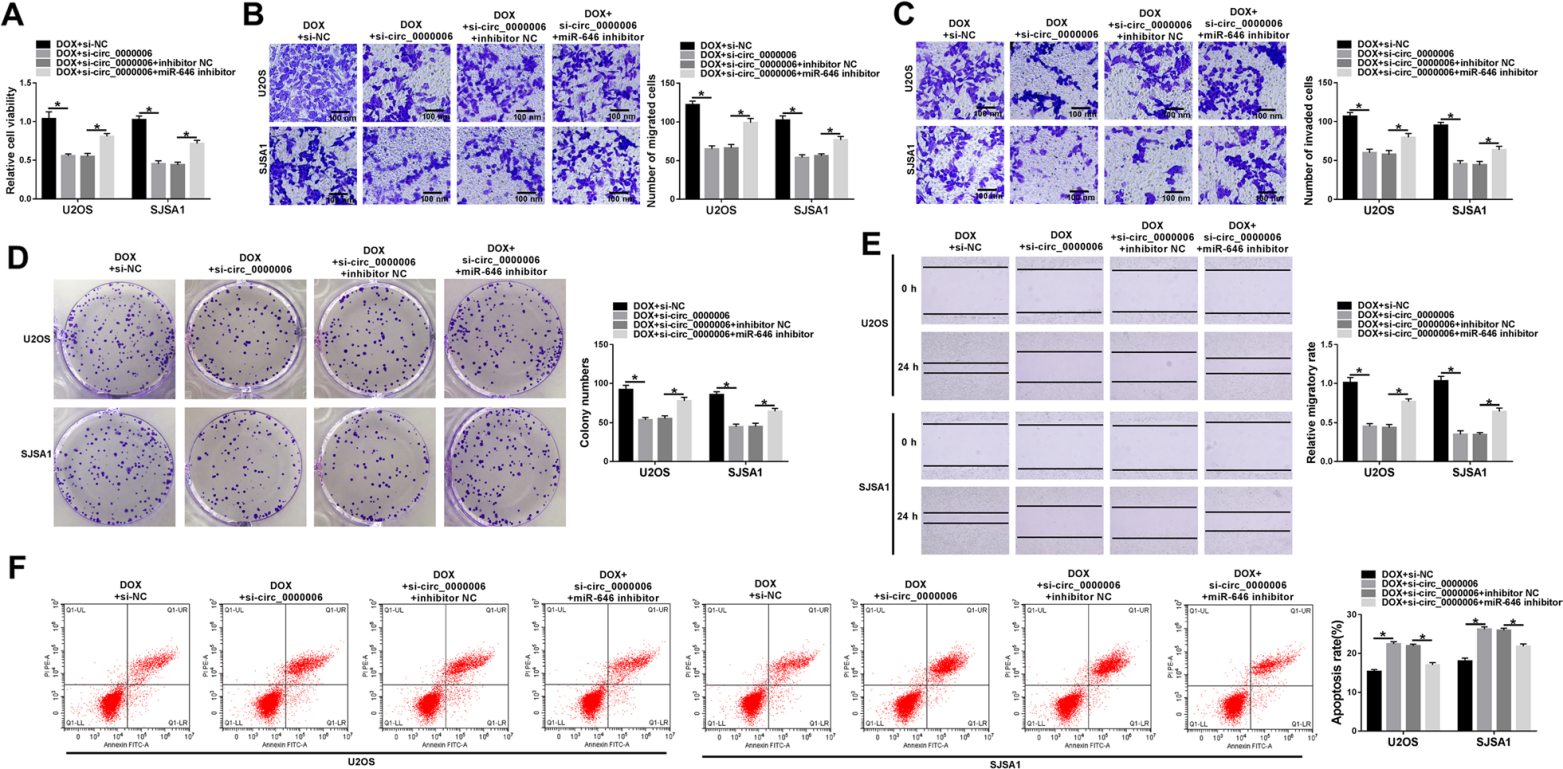
Circ_0000006 silencing expedited the effects of DOX exposure on OS development via associating with miR-646. **A** Cell viability assay was employed to determine the effects between circ_0000006 knockdown and miR-646 inhibitor on cell viability under DOX treatment in U2OS and SJSA1 cells. **B**, **C**, **E** Transwell migration and wound-healing assays and transwell invasion assay were performed to illustrate the influences between circ_0000006 knockdown and miR-646 depletion on cell migration and invasion under DOX treatment, respectively, in U2OS and SJSA1 cells. **D** The effects between circ_0000006 knockdown and miR-646 inhibitor on cell colony-forming ability after DOX exposure were unveiled by cell colony formation assay in U2OS and SJSA1 cells. **F** Flow cytometry assay was carried out to disclose the impacts between circ_0000006 repression and miR-646 inhibitor on cell apoptosis under DOX exposure in U2OS and SJSA1 cells. *P < 0.05

Whether circ_0000006 regulated DOX sensitivity by sponging miR-646 in OS progression was determined in this part. Cell viability assay illustrated that circ_0000006 knockdown repressed cell viability under DOX treatment, whereas this effect was restored by miR-646 inhibitor in U2OS and SJSA1 cells (Fig. 5A). Circ_0000006 silencing inhibited cell migration and invasion after DOX exposure, respectively, in U2OS and SJSA1 cells; however, these impacts were abolished by miR-646 inhibitor (Fig. 5B, C, E). In addition, the colony-forming ability of U2OS and SJSA1 cells was repressed by circ_0000006 depletion after DOX treatment, but this impact was restrained by miR-646 inhibitor (Fig. 5D). Furthermore, the apoptosis rate of U2OS and SJSA1 cells was improved by circ_0000006 repression under DOX treatment, and miR-646 inhibitor relieved this influence (Fig. 5F). Overall, circ_0000006 knockdown facilitated DOX sensitivity by binding to miR-646 in OS process.

### MiR-646 bound to BDNF in U2OS and SJSA1 cells

**Fig. 6:**
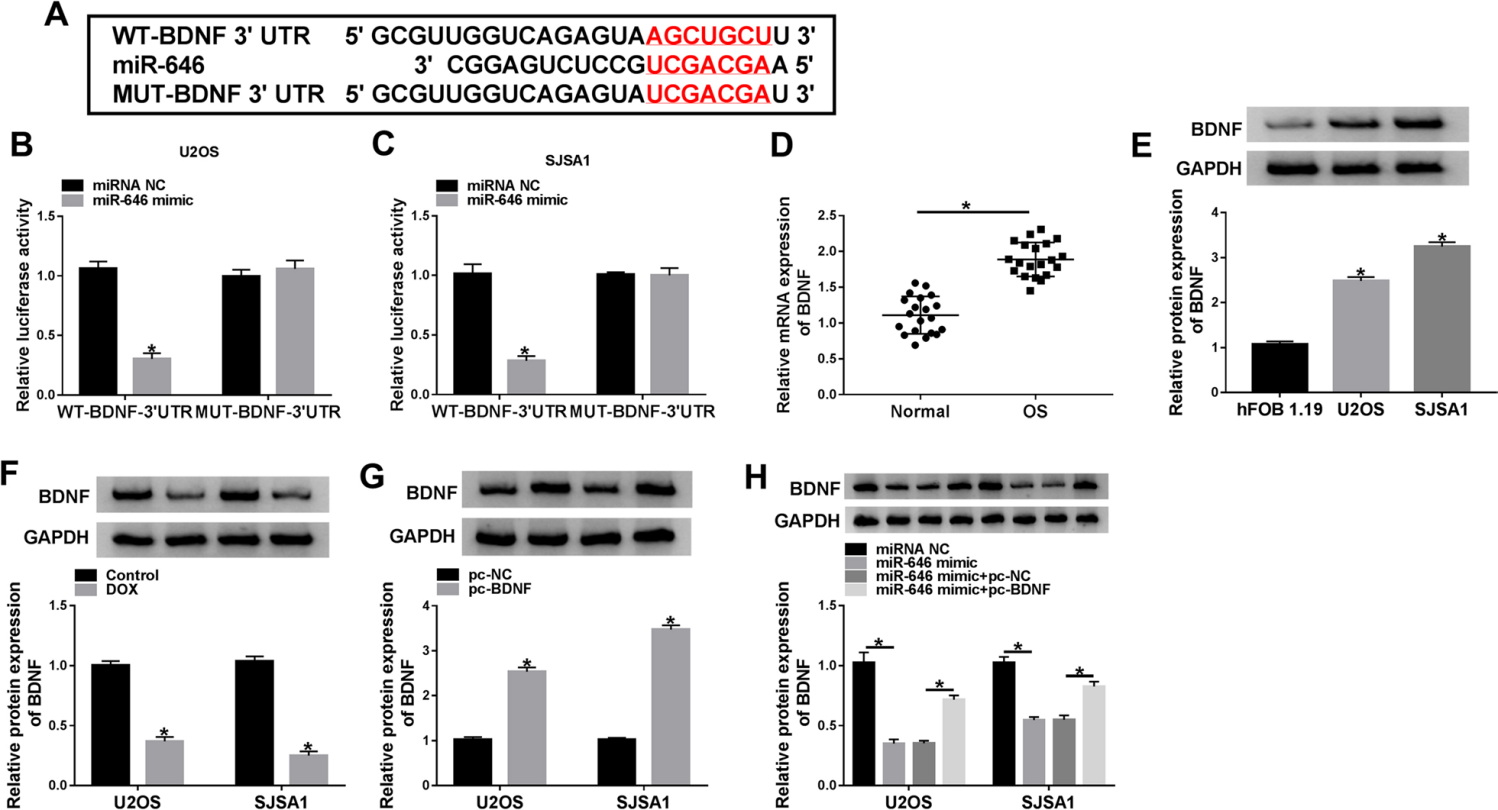
MiR-646 was associated with BDNF in U2OS and SJSA1 cells. **A** Targetscan online database was used to predict the binding sites between miR-646 and BDNF 3′UTR. **B**, **C** Dual-luciferase reporter assay was performed to detect luciferase activities in U2OS and SJSA1 cells. **D**, **E** QRT-PCR and western blot assays were carried out to reveal the mRNA and protein expression of BDNF in OS and normal bone tissues and hFOB 1.19, U2OS and SJSA1 cells, respectively. **F** The effect of DOX treatment on BDNF protein expression was unveiled by western blot in U2OS and SJSA1 cells. **G** The efficiency of BDNF overexpression was determined by western blot in U2OS and SJSA1 cells. **H** The influences between miR-646 mimic and BDNF overexpression on BDNF protein expression were presented by western blot. *P < 0.05

The gene with the ability to bind to miR-646 was further explored. Targetscan online database showed that BDNF 3′UTR contained the binding sites of miR-646 (Fig. 6A). Dual-luciferase reporter assay illustrated that luciferase activity was notably inhibited after WT-BDNF 3′UTR and miR-646 mimic co-transfection in U2OS and SJSA1 cells, whereas there was no prominent variation in MUT-BDNF 3′UTR and miR-646 mimic group (Fig. 6B, C). It was found that BDNF expression was dramatically upregulated in OS tissues and U2OS and SJSA1 cells when compared with control groups, respectively (Fig. 6D, E). Subsequently, the effect of DOX treatment on BDNF expression was unveiled. Western blot analysis illustrated that DOX exposure dramatically repressed the protein expression of BDNF in U2OS and SJSA1 cells (Fig. 6F). Furthermore, western blot was employed to determine the efficiency of BDNF overexpression, and result showed that BDNF protein expression was markedly upregulated by pc-BDNF (Fig. 6G). Western blot also showed that miR-646 mimic dramatically inhibited BDNF protein expression, whereas this effect was restored by BDNF overexpression (Fig. 6H). These results illustrated that miR-646 might modulate DOX sensitivity by binding to BDNF in OS process.

### MiR-646 enhanced DOX-mediated OS process via binding to BDNF

**Fig. 7:**
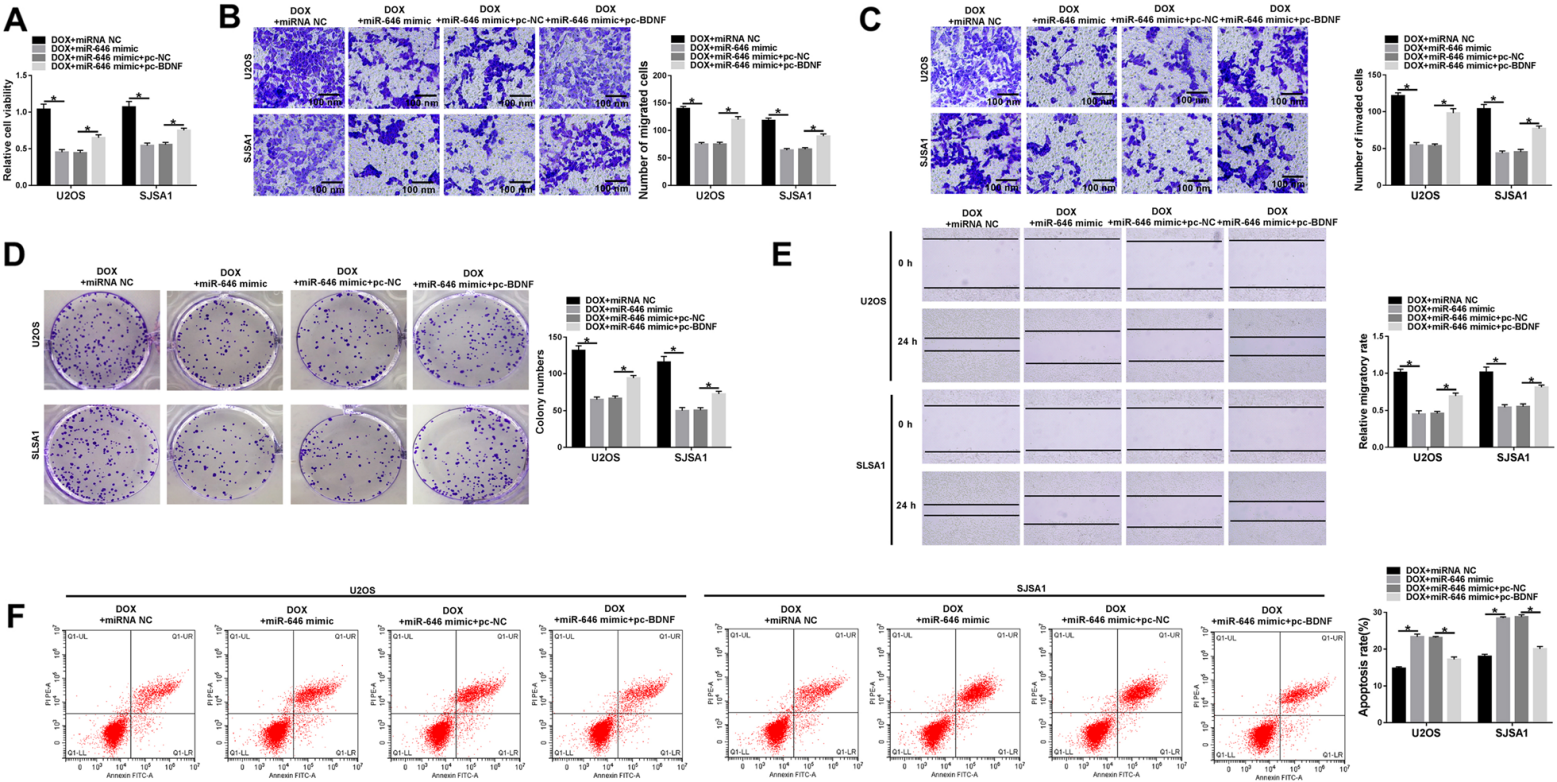
MiR-646 mimic facilitated OS sensitivity to DOX through binding to BDNF. **A** Cell viability assay was performed to determine the effects between miR-646 mimic and BDNF overexpression on cell viability after DOX exposure in U2OS and SJSA1 cells. **B**, **C**, **E** Transwell migration and wound-healing assays and transwell invasion assay were performed to unveil the impacts between miR-646 mimic and enforced BDNF expression on cell migration and invasion, respectively, under DOX treatment in U2OS and SJSA1 cells. **D** The effects between miR-646 and BDNF overexpression on cell colony-forming ability after DOX exposure were revealed by cell colony formation assay in U2OS and SJSA1 cells. **F** Flow cytometry analysis was carried out to explain the influences between miR-646 and BDNF on the apoptosis of U2OS and SJSA1 cells after DOX treatment. *P < 0.05

The underneath effects of miR-646 on DOX sensitivity were further studied. Cell viability assay exhibited that miR-646 mimic inhibited cell viability under DOX exposure in U2OS and SJSA1 cells, whereas this inhibitory effect was attenuated by BDNF overexpression (Fig. 7A). Then, we found that cell migratory and invasive abilities were suppressed by miR-646 mimic, respectively, after DOX exposure; however, these impacts were restrained after pc-BDNF transfection (Fig. 7B, C, E). Furthermore, miR-646 mimic repressed the colony-forming ability of U2OS and SJSA1 cells after DOX treatment; however, BDNF overexpression abolished this impact (Fig. 7D). Flow cytometry analysis demonstrated that enforced BDNF expression attenuated the promoting effect of miR-646 mimic on cell apoptosis after DOX treatment in U2OS and SJSA1 cells (Fig. 7F). Therefore, miR-646 mimic mediated DOX sensitivity via associating with BDNF in OS development.

### Circ_0000006 knockdown repressed BDNF expression via sponging miR-646 under DOX treatment in OS cells

**Fig. 8:**
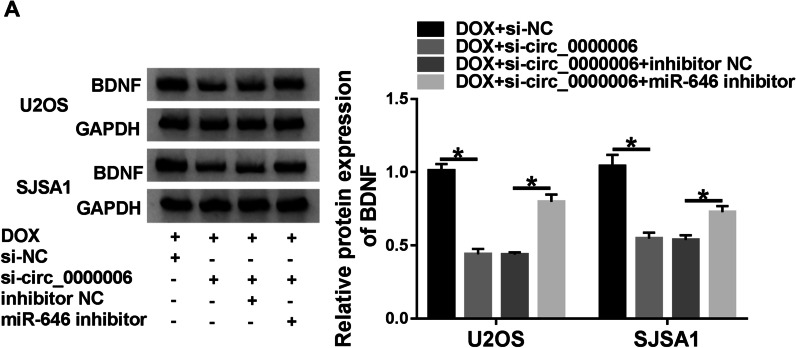
Circ_0000006 absence suppressed BDNF expression by binding to miR-646 after DOX treatment in OS cells. **A** Western blot was employed to manifest the effects between circ_0000006 depletion and miR-646 inhibitor on BDNF protein expression under DOX treatment in U2OS and SJSA1 cells. *P < 0.05

The effects between circ_0000006 knockdown and miR-646 inhibitor on BDNF expression under DOX exposure were further disclosed. Western blot analysis showed that circ_0000006 silencing repressed BDNF protein expression after DOX exposure in U2OS and SJSA1 cells, whereas miR-646 inhibitor restrained this effect (Fig. 8A). These data testified that circ_0000006 knockdown could modulate BDNF expression by associating with miR-646 under DOX exposure in OS cells.

### Circ_0000006 knockdown repressed tumor growth in vivo

**Fig. 9:**
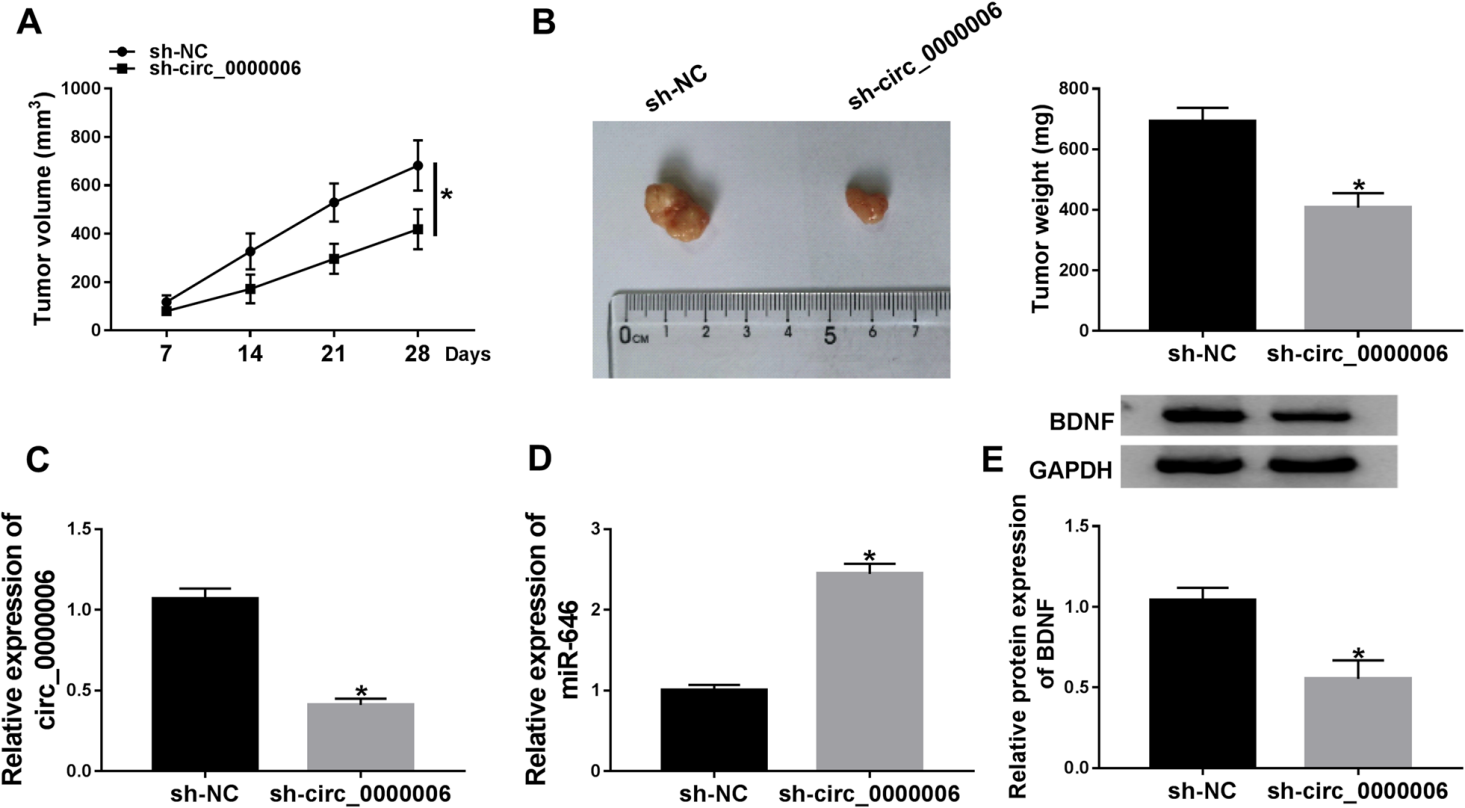
Circ_0000006 knockdown repressed tumor growth by upregulating miR-646 and downregulating BDNF expression in vivo. **A** The effect of circ_0000006 silencing on tumor volume was determined. **B** The effects of circ_0000006 knockdown on tumor size and weight were revealed in vivo. **C** The efficiency of circ_0000006 knockdown was detected by qRT-PCR in vivo. **D** The effect of circ_0000006 depletion on miR-646 expression was illustrated by qRT-PCR in vivo. **E** Western blot was performed to investigate the influence of circ_0000006 repression on the protein expression of BDNF in vivo. *P < 0.05

The effects of circ_0000006 silencing on tumor growth in vivo were further studied. Results showed that circ_0000006 absence dramatically inhibited tumor volume and reduced tumor size and weight (Fig. 9A, B). Furthermore, the impacts of circ_0000006 depletion on the expression of miR-646 and BDNF were revealed in vivo. Circ_0000006 expression was primarily manifested after sh-circ_0000006 transfection, and result showed that circ_0000006 expression was dramatically downregulated by sh-circ_0000006 (Fig. 9C). Subsequently, we found that miR-646 expression was obviously upregulated after sh-circ_0000006 transfection in vivo (Fig. 9D). Western blot result showed that BDNF protein expression was distinctly downregulated by circ_0000006 silencing (Fig. 9E). Accordingly, circ_0000006 absence repressed tumor growth by regulating miR-646 and BDNF expression in vivo.

## Discussion

DOX, an adriamycin, is one of anthracycline anticancer drugs [28]. DOX is widely used to treat cancers, including breast cancer, gastric cancer and OS [6, 29, 30]. However, chemoresistance brings serious burden to its clinical application. Therefore, an in-depth investigation about the resistance of OS to DOX is essential. Given the importance of circRNAs in OS progression [17, 31], the study was designed to demonstrate the effects of circ_0000006 on regulating the sensitivity of OS to DOX and underneath mechanism.

In the present study, we found that DOX exposure repressed cell proliferation, migration and invasion, and facilitated cell apoptosis in OS. Subsequently, the impacts of circ_0000006 knockdown on OS process after DOX exposure were unveiled. Data illustrated that circ_0000006 silencing enhanced the inhibitory effects of DOX exposure on cell proliferation, migration and invasion, and facilitated the promoting impact of DOX treatment on cell apoptosis in OS. We also found circ_0000006 expression was upregulated in OS tissues and cells and was inhibited by DOX treatment in OS cells. In addition, circ_0000006 silencing hindered tumor growth in vivo and circ_0000006 functioned as a sponge of miR-646. Our findings illustrated that circ_0000006 knockdown elevated the efficacy of DOX in OS progression.

MiR-646 is associated with OS progression. For example, Liu et al. presented that long non-coding RNA (lncRNA) zinc finger antisense 1 (ZFAS1) expedited cell ability in colony-forming, migration and invasion via binding to miR-646 in OS [32], suggesting miR-646 repressed OS progression. Yang et al. also implicated that miR-646 mimic suppressed the migratory and invasive abilities of OS cells [33]. In our views, miR-646 inhibitor attenuated the inhibitory effects of circ_0000006 knockdown on cell proliferation, migration and invasion under the exposure of DOX in OS, implicating miR-646 inhibited the proliferative, migratory and invasive abilities of OS cells. Our findings were consistent with the above data. Additionally, miR-646 expression was downregulated in OS tissues and cells and was increased by DOX treatment in OS cells. MiR-646 was revealed to induce cell apoptosis and was associated with BDNF in OS cells.

BNDF, a neurotrophic factor, serves key roles in brain development [34]. More and more research indicated that BDNF widely participated in cell proliferation and migration in various diseases [35, 36]. Recently, data unveiled that miR-496 repressed cell proliferation through associating with BDNF in OS cells [37], which implied that BDNF served as a carcinogen in OS progression. Similarly, in this study, we found that enforced expression of BDNF restored the inhibitory effects of miR-646 mimic on cell proliferative and invasive abilities under DOX treatment in OS, meaning BDNF promoted cell proliferation and invasion. Our findings also showed that BDNF expression was higher in OS tissues and cells and was repressed at protein level after DOX exposure. In addition, BDNF repressed cell apoptosis in OS.

However, the effects of circ_0000006 silencing on tumor growth after DOX exposure in vivo were not studied. The limitation will be solved in following study.

Collectively, the expression of circ_0000006 and BDNF was increased in OS tissues and cells and repressed after DOX exposure in OS cells. MiR-646 expression was downregulated in OS tissues and cells, and upregulated after DOX exposure in OS cells. Circ_0000006 knockdown enhanced the suppressive impacts of DOX on cell proliferation, migration and invasion, and facilitated the promoting effect of that on cell apoptosis through miR-646/BDNF axis in OS. Furthermore, circ_0000006 depletion restrained tumor growth in vivo. Our findings provide a new mechanism for studying DOX sensitivity in OS progression.

## Data Availability

All data generated or analyzed during this study are included in this article.

## Abbreviations

OS: Osteosarcoma
DOX: Doxorubicin
BDNF: Brain-derived neurotrophic factor
